# Miniaturized multi-sensor loggers provide new insight into year-round flight behaviour of small trans-Sahara avian migrants

**DOI:** 10.1186/s40462-018-0137-1

**Published:** 2018-10-02

**Authors:** Felix Liechti, Silke Bauer, Kiran L. Dhanjal-Adams, Tamara Emmenegger, Pavel Zehtindjiev, Steffen Hahn

**Affiliations:** 1 Swiss Ornithological Institute, Department of Bird Migration, Seerose 1, 6204 Sempach, Switzerland; 2 Institute of Biodiversity & Ecosystem Research, Bulgarian Academy of Sciences, Sofia, Bulgaria

**Keywords:** Biologging, Migration, Flight, Timing, Altitude, Activity, Eurasian hoopoe, Great reed warbler

## Abstract

**Background:**

Over the past decade, the miniaturisation of animal borne tags such as geolocators and GPS-transmitters has revolutionized our knowledge of the whereabouts of migratory species. Novel light-weight multi-sensor loggers (1.4 g), which harbour sensors for measuring ambient light intensity, atmospheric pressure, temperature and acceleration, were fixed to two long-distance migrant bird species - eurasian hoopoe (*Upupa epops*) and great reed warbler (*Acrocephalus arundinaceus*). Using acceleration and atmospheric pressure data recorded every 5 and 30 min, respectively, we aimed at reconstructing individual diurnal and seasonal patterns of flight activity and flight altitude and thereby, at describing basic, yet hitherto unknown characteristics of migratory flight behaviour. Furthermore, we wanted to characterise the variability in these migration characteristics between individuals, species and migration periods.

**Results:**

The flight duration from breeding to sub-Saharan African non-breeding sites and back was more variable within than between the species. Great reed warblers were airborne for a total of 252 flight hours and thus, only slightly longer than eurasian hoopoes with 232 h. With a few exceptions, both species migrated predominantly nocturnally - departure around dusk and landing before dawn. Mean flight altitudes were higher during pre- than during post-breeding migration (median 1100 to 1600 m a.s.l.) and flight above 3000 m occurred regularly with a few great reed warblers exceeding 6000 m a.s.l. (max. 6458 m a.s.l.). Individuals changed flight altitudes repeatedly during a flight bout, indicating a continuous search for (more) favourable flight conditions.

**Conclusions:**

We found high variation between individuals in the flight behaviour parameters measured – a variation that surprisingly even exceeded the variation between the species. More importantly, our results have shown that multi-sensor loggers have the potential to provide detailed insights into many fundamental aspects of individual behaviour in small aerial migrants. Combining the data recorded on the multiple sensors with, e.g., remote sensing data like weather and habitat quality on the spatial and temporal scale will be a great step forward to explore individual decisions during migration and their consequences.

**Electronic supplementary material:**

The online version of this article (10.1186/s40462-018-0137-1) contains supplementary material, which is available to authorized users.

## Background

Over the past decade, the miniaturisation of animal borne tags such as geolocators and GPS-transmitters has revolutionized our knowledge of the whereabouts of migratory species. Although we now know where and when many bird species migrate, we still know very little about their behaviour during migration. For instance, important characteristics like how often they engage in sustained flights or land to stop-over, whether they exclusively migrate during day or night, and which heights they choose for migratory flights, are still hardly known.

Knowing the year-round movement patterns is a prerequisite for investigating individual life cycles [1]. To study year-round energy expenditure, individual behaviour in relation to environmental conditions, or carry over effects, not only requires knowledge on where an individual is, but also on how and when it is being active and moves between different locations. Especially in migratory birds, movement patterns are key factors of the annual cycle. In recent years, large birds like seabirds and raptors have been equipped with tags that allow positioning with high resolution (e.g. [2, 3], but also monitor flight altitudes and even behavioural aspects by acceleration sensors [4]. However, the overall weight of these tags has limited their applicability to birds weighing more than 100 g. With the development of miniaturized light-level geolocators [5], the number of studies tracking small passerines and near-passerines has greatly increased (e.g. [6–13]. Light-level geolocators allow the estimation of movement and stationary periods along the annual cycle [14], but provide no insight into the actual flight behaviour.

To date, information on the behaviour of free flying individual small birds, like flight altitude, diurnal pattern of flight time or flight speed and flight direction was only available from radar tracking (e.g. [15–27] and telemetry studies (e.g. [28–31]. With radar, however, species identification is rarely possible and behavioural data can only be collected over short distances (in the best case a few tens of kilometres), with virtually nothing else being known about the tracked individual. Telemetry recordings have been performed by following single birds by plane for several hours or by a network of fixed ground base stations, providing the temporal pattern of passage for a specific area – but again, this only provides information on behaviour over short spatial and temporal scales.

Newly developed miniaturized multi-sensor loggers monitor light for geolocation, and simultaneously record acceleration and air pressure [32]. Analysing acceleration and air pressure at temporal resolutions of minutes sheds light on many aspects of individual behaviour throughout the year – such as daily patterns of categorized behaviour like flying, foraging and resting, and altitudes during flight. The aim of this study is to reveal basic but hitherto unknown migration characteristics in two trans-Saharan migrants, the great reed warbler (*Acrocephalus arundinaceus*) and the eurasian hoopoe *(Upupa epops)*, to illustrate the potential of these new devices. We explore and compare characteristics of migratory behavior of the two species, e.g. it is still discussed whether the eurosiann hoopoe is diurnal or nocturnal migrant [33, 34], while the great reed warbler is generally considered to migrate a night. According to the morphological differences between the two species, we hypothesize that great reed warblers would travel more efficiently than hoopoes, which might be disadvantaged by their broad wings resulting in the well-known fickle flight style. Wing-loading is about 60% higher in the warbler compared to the hoopoe, whereas the aspect ratio of the wing (narrowness) is about 30% higher in the warbler [35]. Higher wing loading is related to higher airspeeds in birds [21], whereas low wing loadings and broad wings produce more lift. Therefore, we expect great reed warbles to fly faster and having longer flight bouts than hoopoes resulting in higher migration speeds, whereas hoopoes would expect to have higher climb rates. In particular, we compare the seasonal pattern of flight bouts, diurnal take-off and landing times, mean and maximum flight altitudes and rates of climb of the two species.

## Methods

### Species and study sites

The eurasian hoopoe is a medium-sized bird, 25–32 cm long, with a 44–48 cm wingspan and a body mass of 46-89 g [33]. We applied 19 multi-sensor loggers (GDL3-PAM) on adult breeders from a population that has been studied since 15 years [36, 37]. The study site is located in an inner-alpine valley in south-western Switzerland (46°14′N 7°22′E).

The great reed warbler measures about 16–21 cm in length, 25–30 cm in wingspan and weighs 22–38 g [38]. The study was done in Biological Station Kalimok (Institute of Biodiversity and Ecosystem Research at Bulgarian Academy of Sciences) located in north-eastern Bulgaria (44°00’ N, 26°26′ E). We equipped 70 adults with the multi-sensor loggers. Studies on this population have taken place since 2005 [39, 40]. The study site is situated in reed beds of a former fish pond area associated with the Danube river.

Both, eurasian hoopoes and great reed warblers are long-distance migrants that migrate from their European breeding grounds to the western Sahel region [6, 41] and to central and eastern Africa [42, 43], respectively. Both species are well known to use flapping flight with no indication of soaring flight [15]. Thus, they are well suited for a comparative study of flight behaviour using accelerometers.

### Multi-sensor loggers

The multi-sensor loggers (i.e. GDL3-PAM) were developed and produced by the Swiss Ornithological institute in cooperation with the Bern University of Applied Sciences. The loggers consist of sensors for measuring ambient light intensity, air pressure, acceleration, temperature and magnetic field. The average weight of a logger in our study was 1.4 g (range 1.3–1.45 g) including battery, coating and leg loop harness, corresponding to 2.2% of mean body mass for a hoopoe and 4.5% for a great reed warbler. Former studies have shown no deleterious effects of the loggers on return rate and reproductive success for hoopoes [44]. Recapture rate of eurasian hoopoes was 26% (5 of 19) for the birds equipped with loggers compared to 18% (12 of 65) for the control group (ringed only birds). Recapture rates of great reed warblers equipped with loggers was 20% (14 of 70), compared to 13% of the controls (5 of 38). One of the great reed warblers equipped with a logger returned without a logger.

Recording intervals of GDL3-PAM loggers can be customized in accordance to a particular study focus. As we aimed for a year-round monitoring of behaviour and movements, while simultaneously optimising battery life and memory usage, we chose measurement intervals of 5 min for light intensity, 30 min for air pressure and temperature recordings and 5 min for acceleration.

### Activity measurements

The accelerometer sensor recorded acceleration along the Z-axis every 5 min for 3.2 s with a frequency of 10 Hz [45]. An on-board algorithm calculated the mean of the 32 values representing the relative position of the body axis with respect to the horizontal plane (pitch), and the sum of the absolute differences between consecutive points (31 values) representing the relative activity (Additional file 1: Figure S1). Field tests with continuous acceleration data (10 Hz) had shown that by using these compressed data, flapping flight can be separated reliably from other activities [45].

**Fig. 1:**
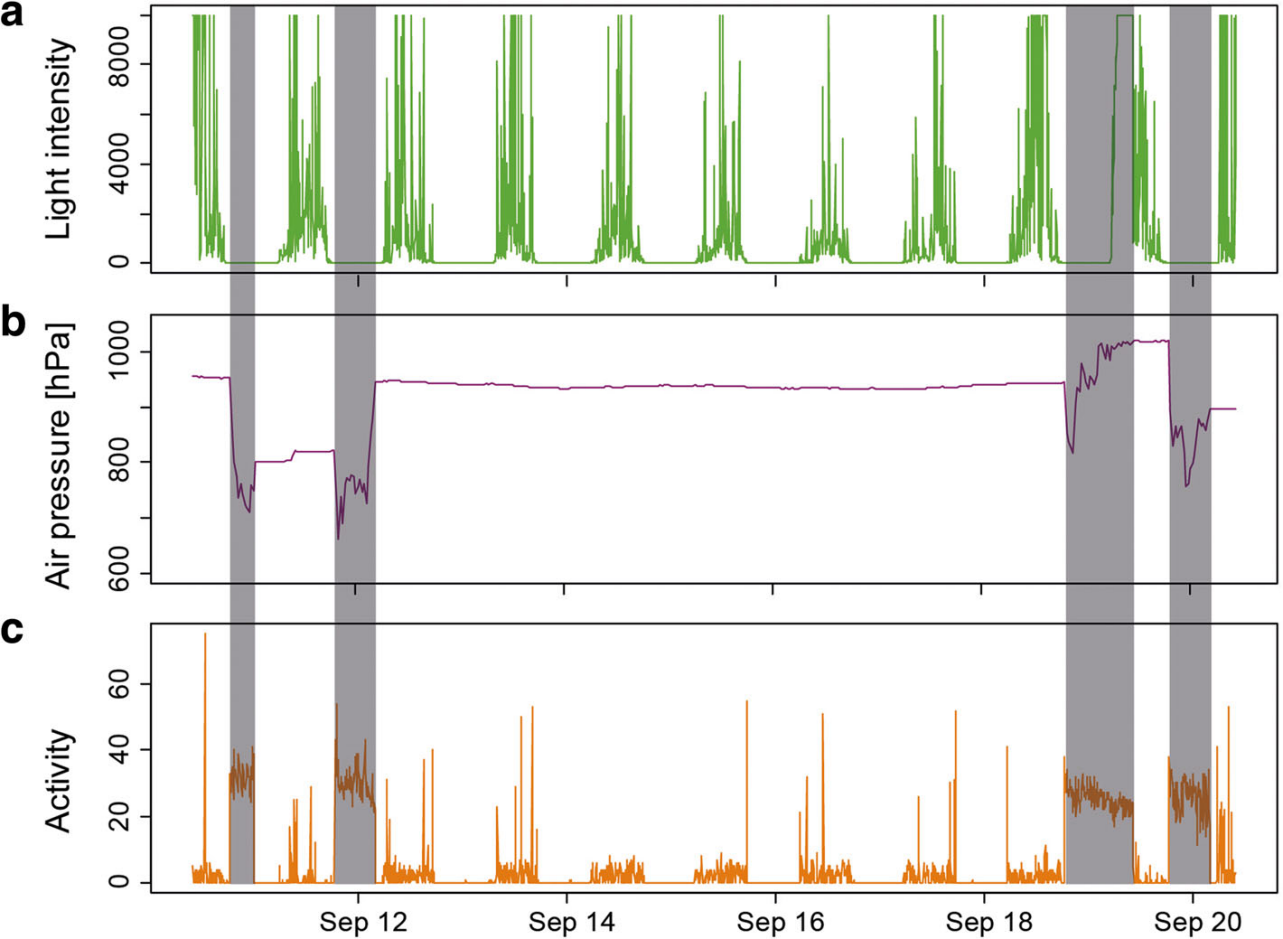
Example of raw data time series of (**a**) light intensity (green), (**b**) air pressure (pink) and (**c**) activity (orange). Activity is derived from accelerometer data and processed on board (see methods). Examples of single flight bouts are marked by shaded areas. Y-scales for light and activity show arbitrary raw values as recorded by the sensors

Data on activity derived from accelerometry is suitable to determine phases of flight activity, because both species exclusively use flapping flight for migration (Fig. 1). We developed an automated algorithm to differentiate between three categories of behaviour (flight, other activities and resting). Resting was defined by an activity level of zero. For each individual tag we revealed the first local minimum from the frequency distribution of the activity recordings. This minimum was assigned as the threshold between flight and other activities (for details see Additional file 1: Figure S2). Finally, flight bouts were defined if at least three consecutive activity recordings were classified as flight, which corresponds to a (minimum) flight duration of 15 to 19 min.

### Altitude measurements

We used atmospheric pressure measurements to estimate altitudes above sea level based on the hypsometric equation [46], assuming standard atmospheric conditions (formula see Additional file 1). For reliability, we compared pressure measurements from the loggers to measurements from a local MeteoSwiss (Federal office of Meteorology and Climatology) weather station (distance 15 km, 12 m higher above sea level) than the logger site) for 3 days before loggers were deployed. Absolute differences ranged from − 0.7 to 3.8 hPa (*n* = 829). The mean difference of 1.5 hPa corresponds very well to the 12 m height difference. Please note that flight altitudes derived from atmospheric pressure measurements alone, based on standardised atmospheric conditions, can deviate from real height by several tens of meters. Sea level pressure varies according to high and low pressure centres passing through an area but rarely resulting in height differences of more than 200 m (~ 25 hPa). All flight altitudes given here correspond to height above sea level (a.s.l.).

The loggers of one eurasian hoopoe and two great reed warblers stopped recording during the pre-breeding (spring) migration period, and were therefore excluded from the overall and pre-breeding migration analyses.

### Data analyses

Timestamps of all tags were linearly corrected for drift of the internal clocks. Clock-drift was less than 7 min for all, except one logger, which had a drift of 2 h over a period of 309 days. We assigned the times of flight based on the activity level (s. above) and determined the start and the end of single flight bouts for each individual. The proportion of nocturnal and diurnal flight activity was determined by referring to the light intensity data recorded in parallel. We calculated mean and maximum flight altitude for each flight bout. Based on the height difference between 30 min of flight we calculated climb rates, and the sum of all height gains across a flight bout (only positive climbs rates). Short-term changes in air pressure within 3-h are reported to be 1–2 hPa ([47] p. 117). Therefore, we expected errors in climb rate estimates due to changes in weather conditions to be insignificant. We used light intensity data to calculate geographic positions using SGAT (https://github.com/SWotherspoon/SGAT), which is mainly based on threshold based positioning in GeoLight [48]. From the breeding sites and the median positions of the longest residency periods in Africa we calculated great circle distance as an estimate of minimum flight distances. For data processing and statistical analysis we used R-3.3.2 [49]. We applied a general linear mixed-effects model (R-package lme4) to test for differences between the two species and seasons with the individuals as a random factor.

## Results

### Flight durations and distance

The flight duration from breeding sites to sub-Saharan African non-breeding sites and back was more variable within than between the species. Great reed warblers needed between 212 to 369 flight hours, with the greatest differences in flight time between individuals was 75%. In eurasian hoopoes, this difference between individuals was only 20% (Table 1). The median of overall flight time was slightly (8%) smaller, whereas the average number of individual flight bouts was very similar with 45 and 47 flight bouts for great reed warblers and eurasian hoopoes, respectively. Overall, in great reed warblers the cummulative great circle distances between breeding grounds and non-breeding residence areas were 1300 km longer than in eurasian hoopoes (Table 1).

**Table 1:** Overview of flight times and migratory distances per species and migration period. Distances given refer to the great circle route between the specific sites of residency along the annual cycle

Period	Species	N	Flight time [hour]		Great circle distance [km]	
			median	range	median	range
overall	Great reed warbler	11	252	212–319	8886	7262–12,752
	European hoopoe	4	232	216–258	7537	7101–8735
post-breeding (<1.11.15)	Great reed warbler	13	82	63–133	3998	3730–4557
	European hoopoe	5	115	71–133	3769	3550–4367
intra-tropical (1.11.15 - 31.1.16)	Great reed warbler	13	25	3–65	796	0–2256
	European hoopoe	5	0	0	0	0
pre-breeding (> 31.1.16)	Great reed warbler	11	137	76–242	4092	3532–5938
	European hoopoe	4	117	88–146	3769	3550–4367

#### Seasonal pattern of flight activity

**Fig. 2:**
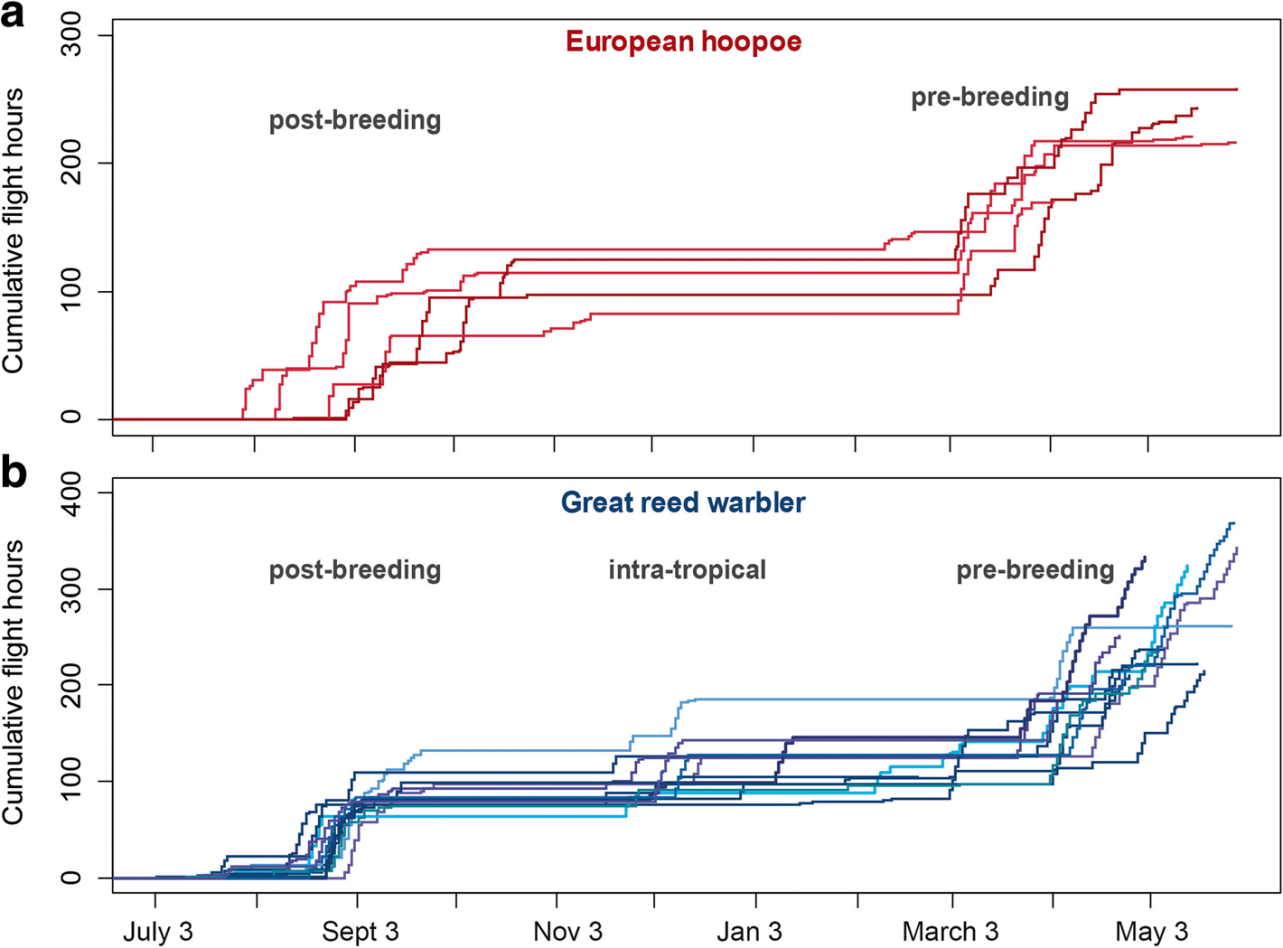
Cumulative hours of flight of eurasian hoopoe (*N* = 5) and great reed wabler (*N* = 13) from July 2015 until April 2016. (**a**) Seasonal activity periods coincide with post- and pre-breeding migration in the hoopoe, while (**b**) reed warblers have an additional activity phase marked as intra-tropical movements

The flight activity over the year clearly revealed a seasonal pattern with peak activity during post- and pre-breeding migration in both species, but also show intra-tropical movement periods for most great reed warblers (Fig. 2). In hoopoes, flight times of post- compared to pre-breeding migration were very similar, while great reed warblers spent considerably more time flying during the pre-breeding migration than they did during both post-breeding migration and intra-tropical migration combined.

**Fig. 3:**
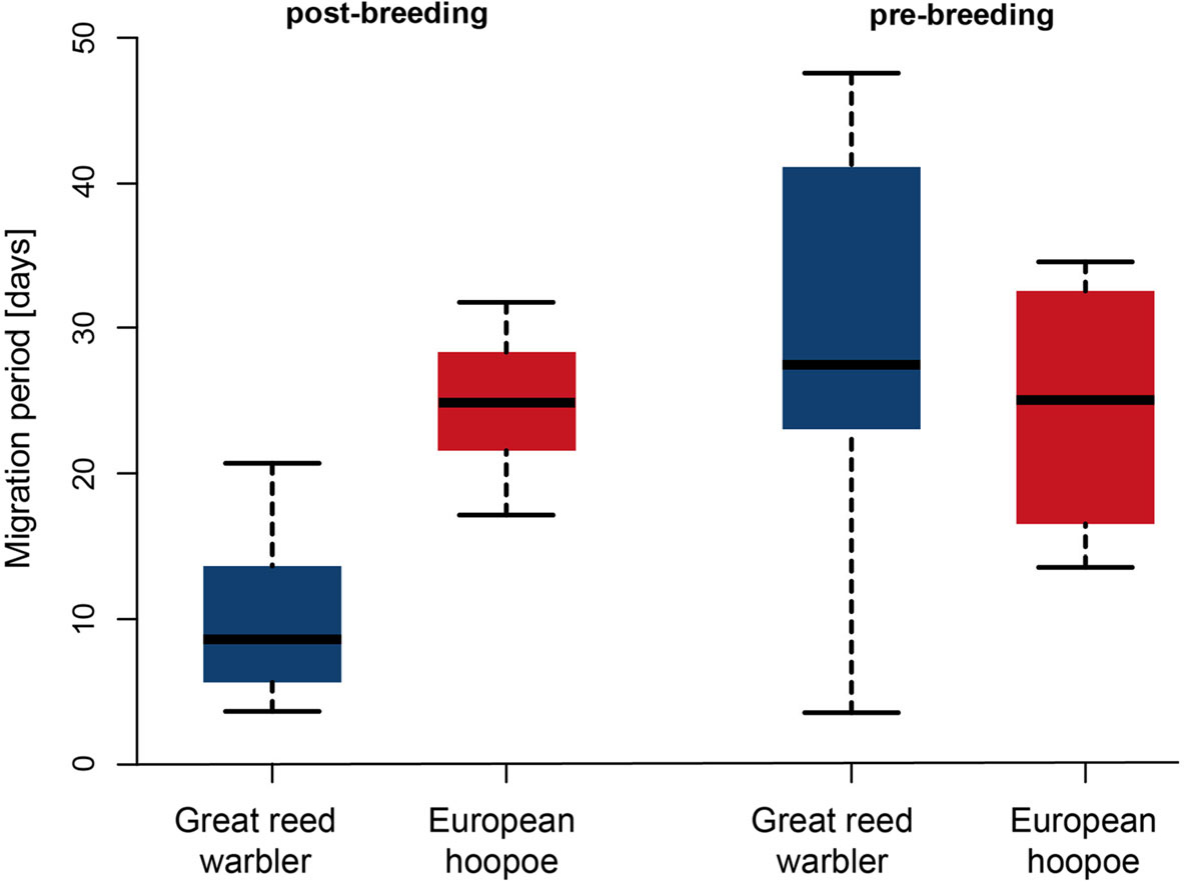
Main period of post- and pre-breeding migration for great reed warbler and eurasian Hoopoe. Shown are the distributions of the shortest time period per individual including 75% of the flight time per migration season. The plots represent median, 50% quantile and range

The temporal pattern of flight activities within the migration periods clearly differed between the species: Long stopovers prolonged the post-breeding migration of hoopoes from August into the end of October, whereas great reed warblers’ flight activity was confined to in the second half of August only. Therefore, the overall duration of post-breeding migration was much shorter in great reed warblers than in hoopoes. To compare the main period of active migration between the species, we determined for each individual the time span that included 75% of the total flight hours per migration season. In hoopoes, the median for the post-breeding time period covered about three weeks, but only one week for great reed warblers (Fig. 3). For the pre-breeding migration, there was no difference between the two species (Figs. 2 and 3).

#### Diurnal flight activity pattern

**Fig. 4:**
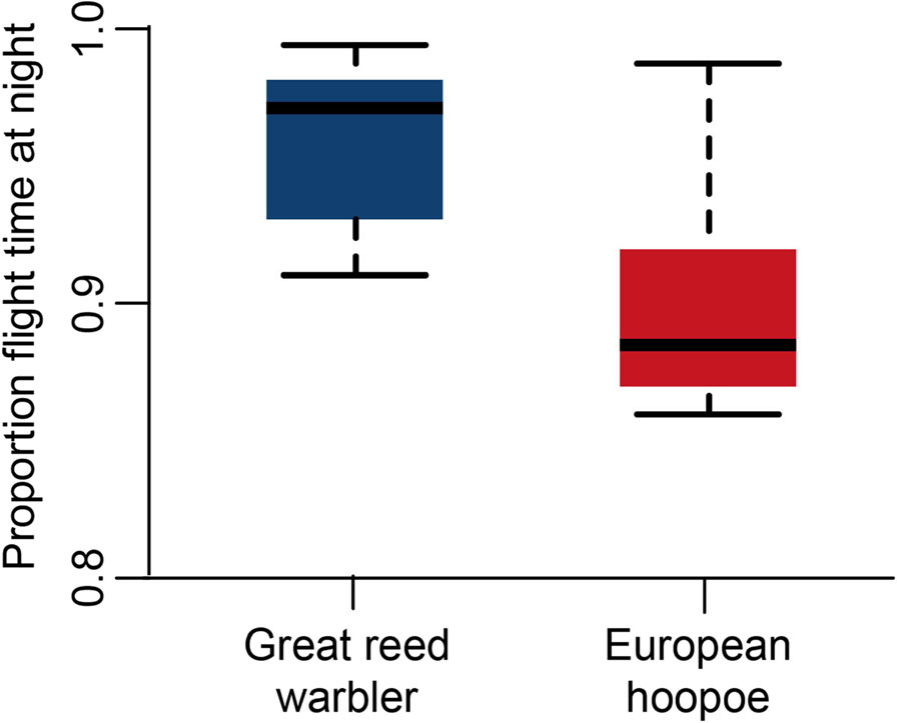
Distribution of the individual proportion of nocturnal flight activity for eurasian hoopoes (red, *n* = 5) and great reed warblers (blue, *n* = 13). Day and night was derived from the tag’s light sensor. The plots represent median, 50% quantile and range

**Fig. 5:**
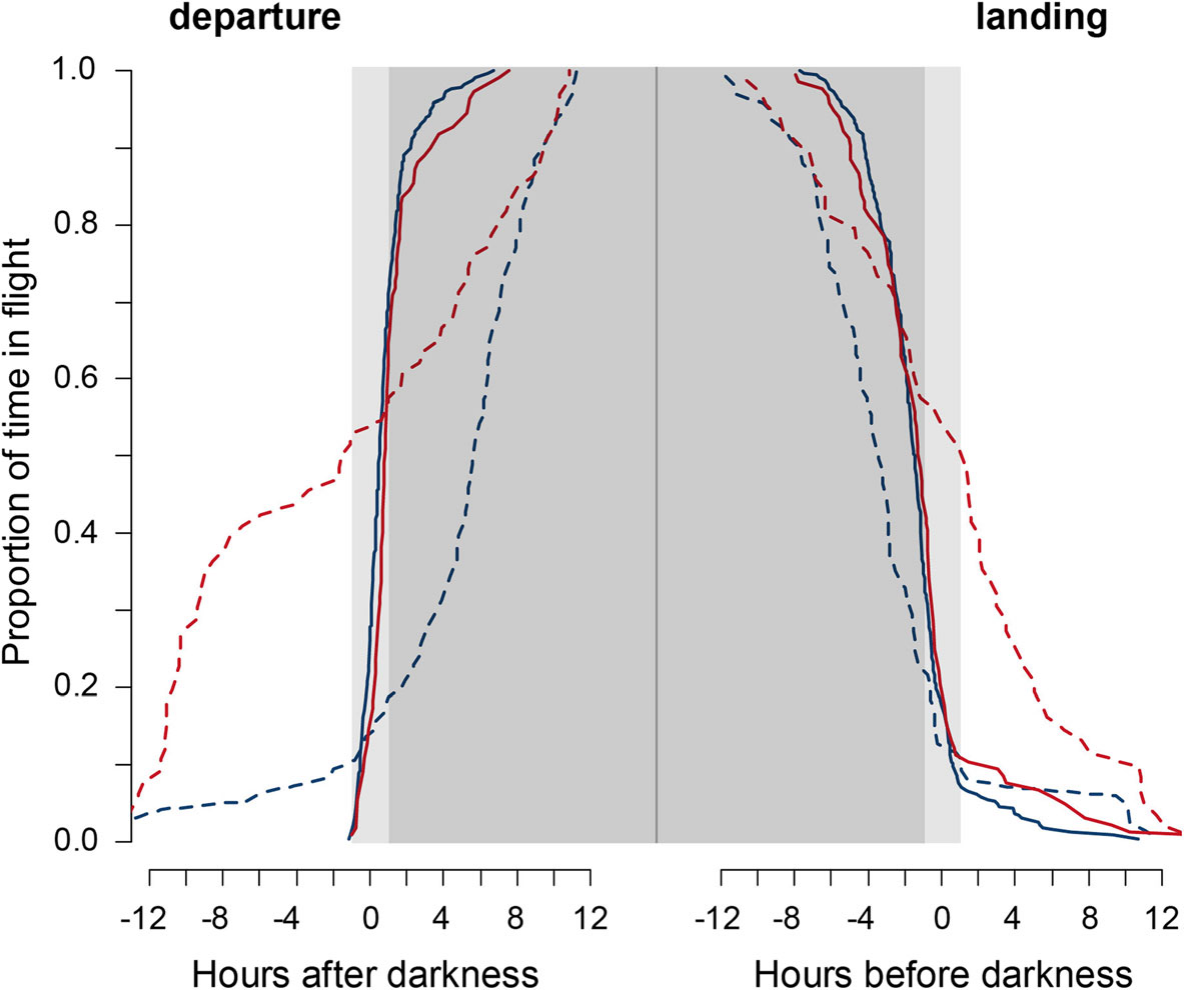
Timing of departure and landing for eurasian hoopoes (red) and great reed warblers (blue), separated into long (> 4 h, solid lines) and short flight bouts (< 1 h, dashed lines). Shown are the cumulative frequencies of departures in relation to dusk and landings in relation dawn. Y = 1 indicates all birds have departed, Y = 0 all birds have landed. Beginning and end of the night (darkness) are derived from the tag’s light sensor. The light shaded area marks an hour before and after darkness, the darker area marks the night

Great reed warblers flew almost exclusively at night (median 97%), whereas in eurasian hoopoes the proportion of nocturnal flights was slightly lower (median 88%; Fig. 4). Departures for flights of > 4 h occurred exclusively in the evening, predominately around dusk (approx. Sun elevation - 3°), with 90% of the departures occurring between 1 h before and 2 h after sunset (both species, Fig. 5, solid lines). Most birds landed before dawn, but landing could also occur during the day in both species. These prolonged flights into the day were more frequent in hoopoes (7% > 12 h, maximum 28 h) compared to great reed warblers (4% > 12 h, maximum 22 h). Short flights of less than 1 h occurring predominantly during migration periods (Fig. 2) were also more frequent in hoopoes (30%) than in warblers (19%). These short flights were initiated irrespective of day or night in hoopoes, while warbles preferred night time also for short flights (Fig. 5, dashed lines).

#### Flight altitudes

**Fig. 6:**
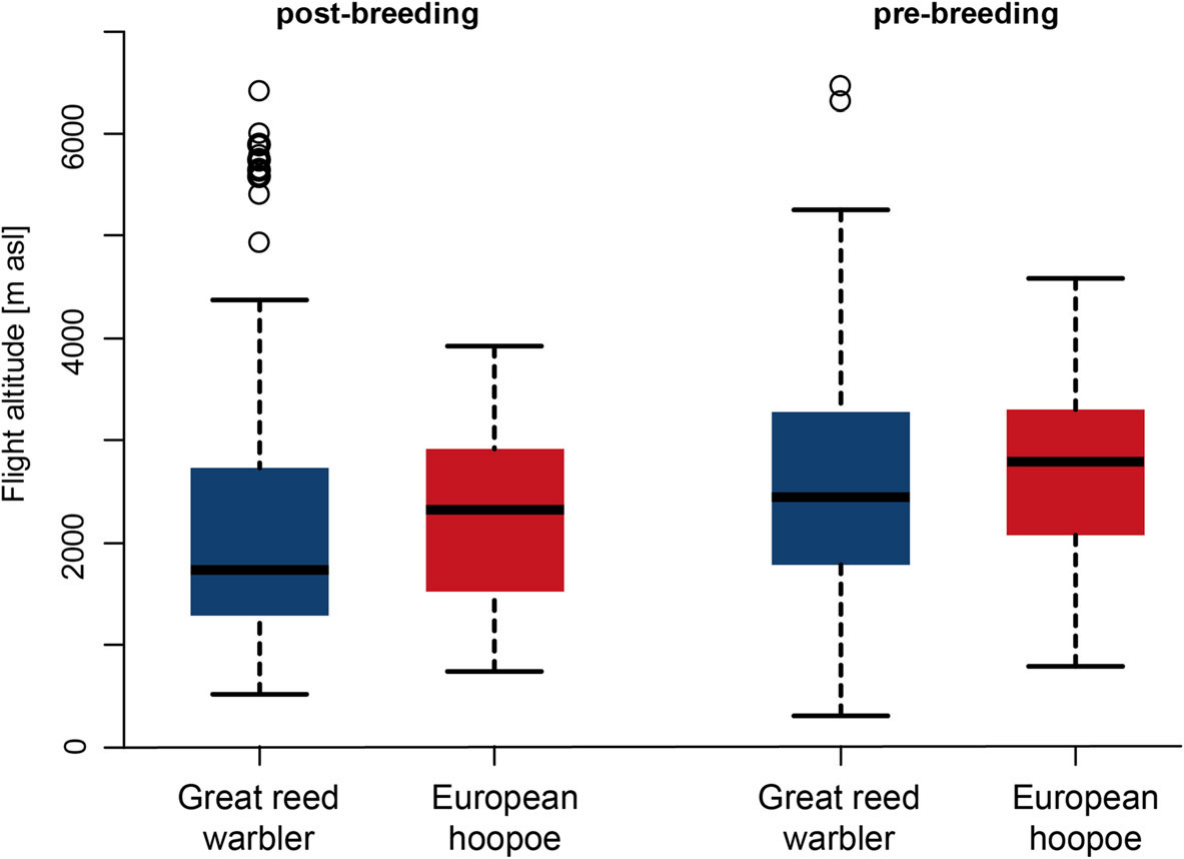
Distribution of the seasonal maximum individual flight altitudes per flight bout of at least 4 h for great reed warblers (blue, *n* = 352) and eurasian hoopoes (red, *n* = 110)

Median flight altitude during post-breeding migration was about 1150 m a.s.l. in both species, and around 1630 m a.s.l. during pre-breeding migration (Additional file 1: Table S1 and Figure S4). All individuals of both species flew at least once above 3000 m a.s.l., and in both species maximum flight altitudes per flight bout were slightly higher during pre-breeding compared to post-breeding migration. Nine out of 13 great reed warblers flew above 5000 m, and 3 of them occasionally even above 6000 m (a.s.l.). Maximum altitude recorded for a great reed warbler was 6458 m a.s.l., and 4584 m a.s.l. for a European hoopoe (Fig. 6). There was a significant difference in mean and maximum flight altitudes between pre- and post-breeding migration (GLM for the mean heights, estimate = 406.7 ± 76.6, t value = 5.3; maximum heights, 460.9 ± 117.6, t value = 3.9), but not between the two species (GLM for the mean heights, 94.2 ± 102.4, 0.9; maximum heights, 71.7 ± 168.3, 0.4).

**Fig. 7:**
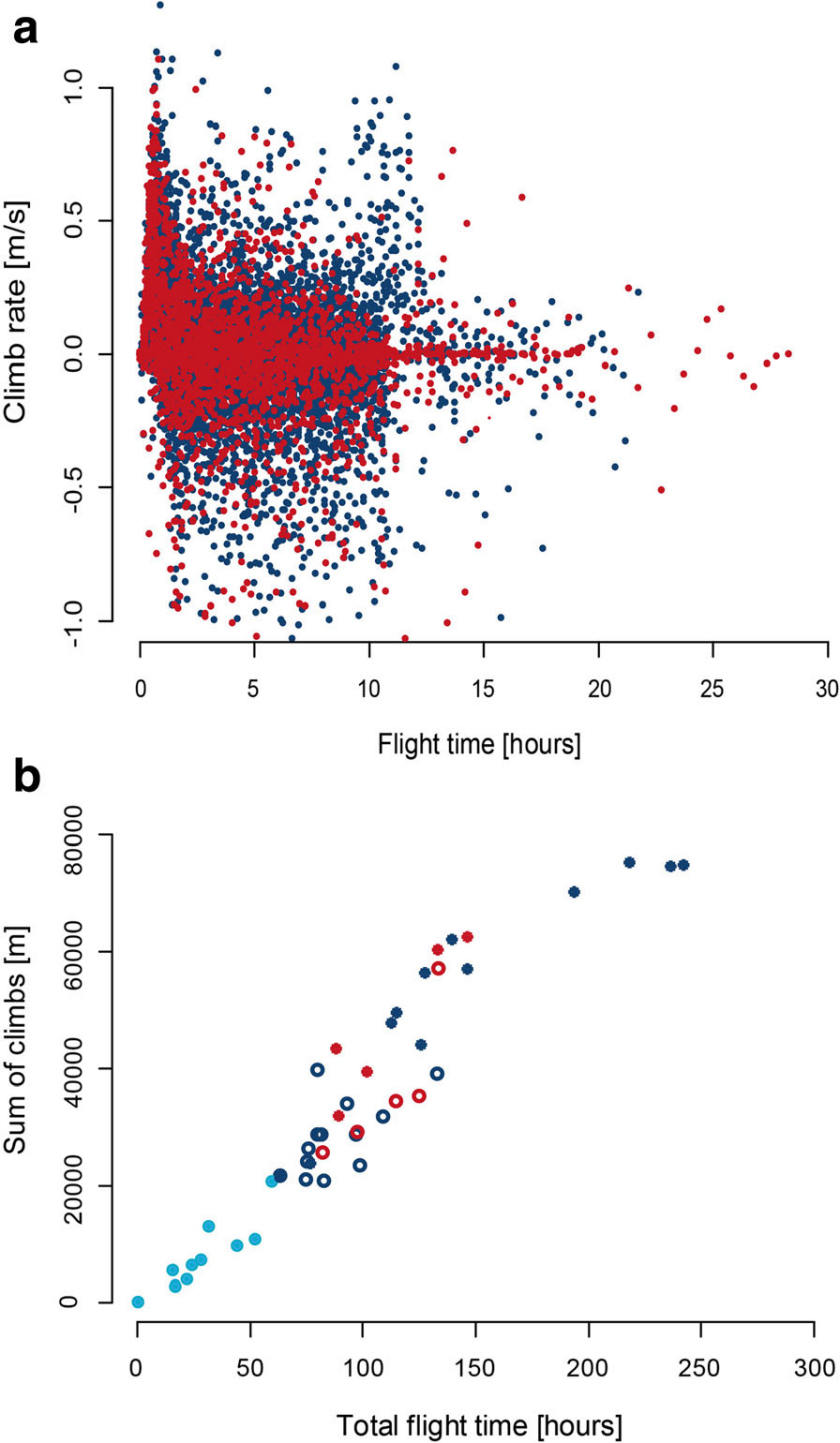
Climbing behaviour of great reed warblers (blue) and european hoopoes (red): **a**) rate of climbing and descending per 30 min flight intervals in relation to the time after departure. A climb rate of 1 m/s corresponds to a height change of 1800 m within 30 min and a change in air pressure of about 190 hPa. The final landing phases (last 30 min) are excluded (reed warbler *n* = 7150, hoopoes *n* = 2448). **b**) sum of climbs for post-breeding (open symbols), intra-tropical (filled light blue) and pre-breeding migration (filled blue/red) in relation to the total individual flight time per migration period

There was a significant relationship of decreasing climb rates with increasing flight duration (Ҳ^2^ = 221.3, *p* < 0.0001, *N* = 4739), which mainly resulted from high climb rates occurring more frequently within the first hour after departure (Fig. 7a). Apart from this initial effect, high climb rates were not related to the time of flight. Climb rates of hoopoes were slightly higher compared to great reed warblers (mean 0.19 m/s vs 0.17 m/s; Ҳ^2^ = 3.7, *p* = 0.053). In great reed warblers, the overall rate of change in flight altitude within 30 min was 159 m (median, 50% range: 54-367 m) and 194 m (55-442 m) in eurasian hoopoes. In great reed warblers the total amount of height changes was 100% larger during pre- than post-breeding migration and 50% larger in hoopoes. However, these height changes were closely related to the individual flight durations per migration period (Fig. 7b). These observations indicate that flight altitude was hardly constant over more than a few dozen minutes.

## Discussion

The data from multisensory loggers provide novel and highly detailed insight into the individual timing of flights and the altitudinal pattern for two morphologically different species. Most strikingly, our study shows that variations in behavioural traits, such as length of flight bouts, flight altitude and migratory speed are larger within than between species for the whole annual cycle.

### Seasonal pattern in flight activity

There is no obvious difference in the flight activity between the two species with respect to total flight hours, number of flight bouts for pre- and post-breeding migration, and also the intra-tropical migration performed by the great reed warbler alone [42] does not account for a consistent difference between the species. On average, the intra-tropical migration represented only 10% of overall flight time and varied considerably between individuals. It is generally assumed that pre-breeding migration is more time constrained than post-breeding migration [50, 51]. In contrast to this assumption, we did not find a difference in the length of post- and pre-breeding migration in hoopoes, and in great reed warblers post-breeding migration was much shorter and much more synchronized than pre-breeding migration. Most likely, the first non-breeding residence site in sub-Saharan Africa may provide temporarily restricted food resources, because most individuals migrate further after about two months [43]. Ephemeral food availability at these sites would increase the need to be there in time, perhaps just after the seasonal rainfalls.

It is unclear why great reed warblers spent considerably more time in flight during the pre-breeding migration, than during both autumn migration and intra-tropical movement. Longer flight times might be caused either by detours or slower ground speeds due to opposing winds. Although pre-breeding migration was more extended than post-breeding migration, due to the additional movements southward during December, one bird returned from its residence area south of the Sahara within one week, flying 74.5 h during 8 consecutive nights (2-18 h per day). Assuming that flight costs relates to 1% of the body mass per hour (Hedenström 2010), a bird would be expected to have lost about 50% (if no refuelling occurred during daytime), which is close to the limit of the range of body mass observed along the migratory flyway [52].

### Diurnal flight activity pattern

For the first time we could quantify the proportion of nocturnal flights in small long distance migrants. The results confirm previous (anecdotal) observations that great reed warblers are predominately nocturnal migrants and clarify the status of eurasian hoopoes as a mainly nocturnal migrant. The regular occurrence of short diurnal flights (Fig. 5), and thus the potential for visual observations during the day, may have led to the ambiguous assessment of the species as a mainly diurnal and occasional nocturnal migrant.

### Flight altitude

Surprisingly, flight altitudes of thousand meters above sea level or more are common in both species, and are not likely indicative of mountain crossings. Indeed, very high altitude flights occur regularly during post- and pre-breeding migration, and are more frequent in great reed warblers than in eurasian hoopoe. This difference may be partly a result of unequal sample sizes. An overview including quantitative radar observations from the Baltic Sea to the Sahara, revealed an upper limit of the 90% quantile to be between 1400 and 2100 m a.s.l. [53]. We found an upper limit of more than 3000 m a.s.l. for both species, which is considerably higher than previously observed flight heights (Additional file 1: Figure S4). Whether this is due to a geographically biased sampling of radar data or due to a different preference of flight altitude by these species, remains an open question. We assume that flights at very high altitude are related to favourable wind conditions, as has been also seen by radar observations [54].

Climbs and descents occur throughout a flight bout, and there was only a slight decrease in climb rates with time in flight. This indicates that flight altitudes are changed throughout a flight bout, either due to crossing mountain ranges and/or to changing wind conditions. Some great reed warblers flying into the day (after 10-12 h of flight) show a considerably increased climb rate leading to very high flight altitudes (Fig. 7a). The overall sum of ascents was considerably larger during pre- than post-breeding migration, which supports the seasonal differences in flight altitudes recorded by radar in the western Sahara [18]. The somewhat higher climb rates recorded in the eurasian hoopoe compared to the great reed warbler confirm the expectations based on their lower wing loadings. The overall sum of ascents makes up about 1% of the total great circle distance covered by an individual bird (see Table 1, Additional file 1: Table S2), and further investigations will be needed to understand the costs and benefits of these vertical movements.

## Conclusion

We could not support our initial hypothesis that eurasian hoopoes with their broader wings and the fickle flight style are less efficient migrants with respect to flight behaviour. Neither flight altitude, the number of flight hours per distance covered nor the lengths of single flight bouts indicated varying flight efficiency between the two species. Our analyses have shown that multi-sensor loggers have the potential to provide insights into many fundamental aspects of individual behaviour in small aerial migrants. Naturally, our analyses are only the tip of the iceberg entailing a range of future analyses. For instance, accuracy in light-based geolocation is heavily affected by shading effects, thus activity patterns can accurately distinguish movement and stationary periods, and air pressure recordings during stop-over periods could be used to narrow down the position of stop-over areas. Furthermore, activity patterns during stop-over or resident periods might provide information on individual habitat use and energy budgets. Combining flight altitudes and locations with environmental/weather data can identify cues for migratory decisions and can significantly help to improve individual movement models. The detailed and long term recordings of data, as presented in this study, are suitable not only for bird migration studies, but also for many other investigations in the field of behavioural research and movement ecology.

## Additional file

Additional file 1: Formula. Calculation of height from pressure recordings. **Table S1.** Overview of mean flight altitudes per species and season. **Table S2.** Overview of the sum of ascents per species and migration period. **Figure S1.** Illustration of recording and data compression of acceleration data. **Figure S2.** Example of the frequency distribution of the activity recordings (definition see Fig. S1) **Figure S3.** Great circle distances between seasonal residence areas in relation to flight time. **Figure S5.** Standardized residual plot of the generalized linear mixed effect model. (DOCX 185 kb)
