# Supplementary material for: Miniaturized multi-sensor loggers provide new insight into year-round flight behaviour of small trans-Sahara avian migrants
Source: Mov Ecol. 2018 Oct 2;6:19. doi: 10.1186/s40462-018-0137-1 (PMC6167888; doi:10.1186/s40462-018-0137-1)
Supplement: Supplementary file 1 — Formula. Calculation of height from pressure recordings. Table S1. Overview of mean flight altitudes per species and season. Table S2. Overview of the sum of ascents per species and migration period. Figure S1. Illustration of recording and data compression of acceleration data. Figure S2. Example of the frequency distribution of the activity recordings (definition see Fig. S1) Figure S3. Great circle distances between seasonal residence areas in relation to flight time. Figure S5. Standardized residual plot of the generalized linear mixed effect model. (DOCX 185 kb) [file 40462_2018_137_MOESM1_ESM.docx]

Supplemental material

**Calculation of height from pressure recordings**

Formula for calculating flight altitude from barometric pressure (p) measured by the data logger. Sea level pressure (p_0_) was set to 1013.25 hPa, and sea level temperature (T) was set to 15°C.

$$h=\frac{\left[ \left( \frac{p_{0}}{p} \right)^{\frac{1}{5.255}}-1 \right]\times\left( T+273.15 \right)}{0.0065}$$

**Tables**

Table S1: Overview of mean flight altitudes per species and season. Mean heights per individual were derived from the means per flight bouts weighted by the duration of the flight bout.

|  |  |  | mean flight altitude [m asl] | | | | |
| --- | --- | --- | --- | --- | --- | --- | --- |
|  |  | N | min | 25% | median | 75% | max |
| overall | Great Reed warbler | 13 | 1245 | 1372 | 1454 | 1545 | 1712 |
|  | European Hoopoe | 5 | 1327 | 1340 | 1385 | 1466 | 1583 |
|  |  |  |  |  |  |  |  |
| post-breeding | Great Reed warbler | 13 | 840 | 1061 | 1174 | 1256 | 1711 |
| <1.11.15 | European Hoopoe | 5 | 937 | 1092 | 1113 | 1402 | 1453 |
|  |  |  |  |  |  |  |  |
| intratropical | Great Reed warbler | 13 | 431 | 798 | 990 | 1359 | 1516 |
| 1.11.15 - 31.1.16 | European Hoopoe | 5 | - | - | - | - | - |
|  |  |  |  |  |  |  |  |
| pre-breeding | Great Reed warbler | 12 | 1346 | 1543 | 1654 | 1857 | 2036 |
| >31.1.16 | European Hoopoe | 5 | 1454 | 1479 | 1610 | 1744 | 1781 |

Table S2: Overview of the sum of ascents per species and migration period. Given are range, 50% quantile and median.

|  | **species** | **sum of climbs [m]** | | | | |
| --- | --- | --- | --- | --- | --- | --- |
|  |  | min | 25% | median | 75% | max |
| **overall** | Great Reed warbler | 73830 | 79673 | 88670 | 104599 | 119731 |
|  | European Hoopoe | 74207 | 87402 | 93754 | 96903 | 100490 |
|  |  |  |  |  |  |  |
| **post-breeding** | Great Reed warbler | 20988 | 23559 | 28790 | 31843 | 39846 |
| **<1.11.15** | European Hoopoe | 16389 | 28435 | 34603 | 35304 | 57024 |
|  |  |  |  |  |  |  |
| **innertropical** | Great Reed warbler | 199 | 3038 | 7191 | 10756 | 22904 |
| **1.11.15 - 31.1.16** | European Hoopoe | 0 | 414 | 827 | 5124 | 9422 |
|  |  |  |  |  |  |  |
| **pre-breeding** | Great Reed warbler | 23907 | 48723 | 57086 | 71318 | 75399 |
| **>31.1.16** | European Hoopoe | 39604 | 42500 | 51935 | 60938 | 62538 |

**Figures:**

Figure S1: Recording and data compression of acceleration data. The graph shows a continuous measurement of 12 seconds with a frequency of 10 Hz. For logging year-round data a sample of 3.2 seconds taken every 5min. and compressed by onboard calculation to achieve the mean and the sum of the absolute differences between consecutive points.

$activity = \sum_{i=1}^{31} {abs(x}_{i}-x_{i+1)}$


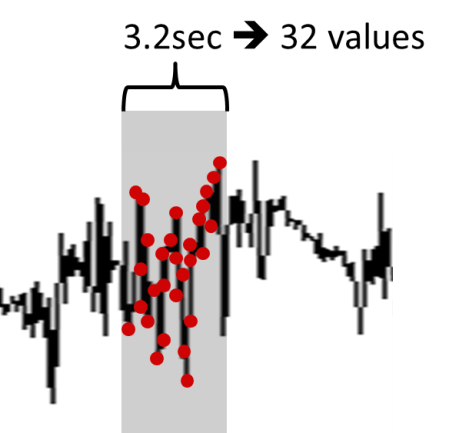


Figure S2: Example of the frequency distribution of the activity recordings (definition see Fig. S1) across the whole sampling period (15.07.2015 – 28.05.2016) for a single tag (14HC). The red line indicates the first minimum, which is taken as the threshold for differentiating between flight and other activities.


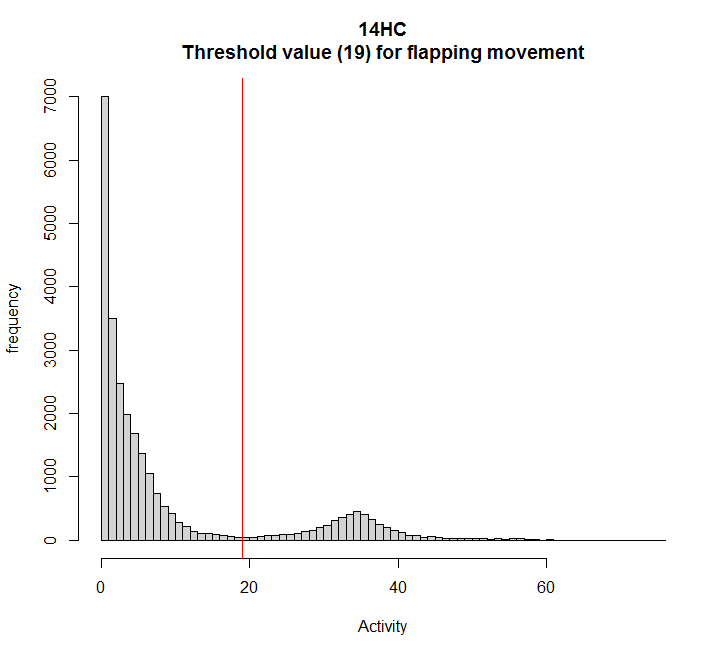


Figure S3: Great circle distances between seasonal residence areas in relation to flight time. Distances are calculated from the median position of the longest staging period within the specific residency.


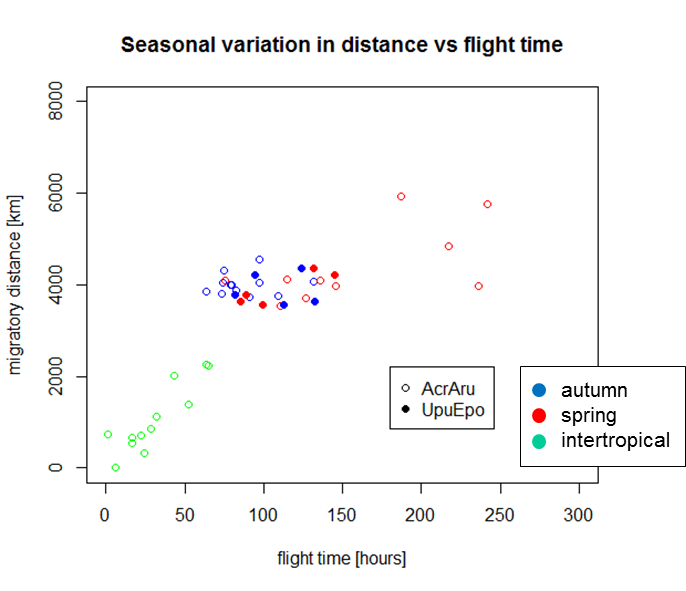


Figure S4: Height distribution of European Hoopes and Great Reed warblers during migration. Shown are the relative frequencies of all individual 30 minutes recordings.


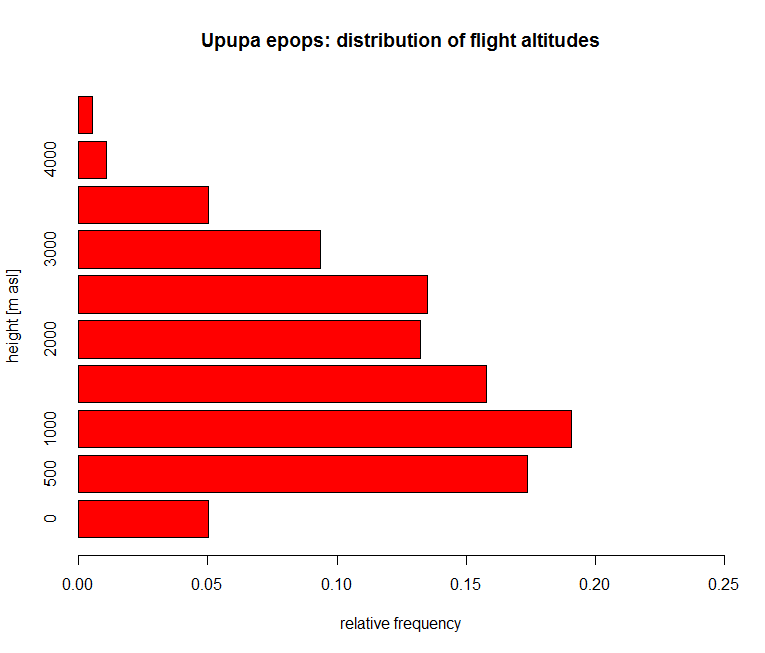

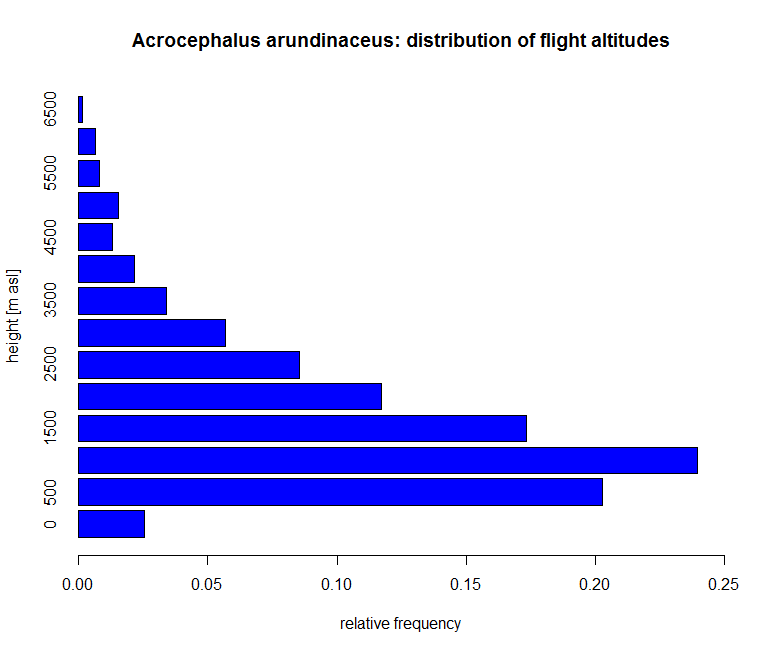


Figure S5: Standardized residual plot of the generalized linear mixed effect model evaluating the difference in climb rates between migratory seasons and species. Model structure: climb-rate ~ time-of-flight + species + (1|species:id).
